# Evaluation of the Effect of Artemisia Absinthium L. Eye-Cream on Infra-Orbital Dark Circle: A Randomized, Double-Blind, Placebo-Controlled Clinical Trial

**DOI:** 10.31661/gmj.v12i.2413

**Published:** 2023-04-26

**Authors:** Hanan Hamdi, Laila Shirbeigi, Mitra Rahimzadeh, Alireza Firooz, Gholamreza Amin, Kazem Mousavizadeh, Arman Zargaran

**Affiliations:** ^1^ Department of Traditional Pharmacy, School of Persian Medicine, Tehran University of Medical Sciences, Tehran, Iran; ^2^ Department of Traditional Medicine, School of Persian Medicine, Tehran University of Medical Sciences, Tehran, Iran; ^3^ Department of Biostatistics and Epidemiology, School of Health, Social Determinants of Health Research Center, Alborz University of Medical Sciences, Karaj, Iran; ^4^ Center for Research & Training in Skins Diseases & Leprosy, Clinical Trial Center, Tehran University of Medical Sciences, Tehran, Iran; ^5^ Department of Pharmacognosy, Faculty of Pharmacy, Tehran University of Medical Sciences, Tehran, Iran; ^6^ Department of Pharmacology, School of Medicine, Iran University of Medical Sciences

**Keywords:** Artemisinin, Persian medicine, Periorbital hyperpigmentation, Infra Orbital Dark circle, Herbal medicine

## Abstract

Background: The relative darkening of the lower eyelid skin, which is often linked with dark circles, may make you seem fatigued and older than your real age. Considering the recommendations in the sources of Persian medicine regarding Artemisia absinthium L., the purpose of this clinical trial is investigating the effectiveness of cream prepared from the aqueous extraction of A.absinthium to remove periorbital dark circles.Materials and Methods: The eye cream is made with 20% of aqueous extract of A.absinthium in the base of the cream. It was standardized based on Artemisinin via HPLC method. For the clinical trial, 60 patients equally enrolled in two drug and placebo groups. Erythema and Pigmentation were evaluated via a mexameter instrument. Results: The cream is standardized, including 1.29±0.02 µg/mg Artemisinin in the product. Finally, 21 and 24 patients reached the end of study in drug and placebo groups, respectively. In these groups, the difference in the mean (SD) DE, DL, Erythema and Melanin factors before and after the research were significant (p0.05). However, the rate of reduction of DE, Erythema, and Melanin and rise of DL is greater in the treatment group than in the placebo group. Furthermore, the mean value of DE and DL factors before the research were significantly different in two groups (p0.001), but after the research did not show a significant difference. The mean value of Erythema factor in the two groups before (p=0.25) and after (p=0.5) did not show a significant difference. The mean value of Melanin after the research between two groups showed a significant difference (p=0.01). Conclusion: The results show that the cream prepared from the herbal composition of Persian medicine improves Infra Orbital Dark circle around the eyes.

## Introduction

Infra Orbital Dark circle (IOD) is identified as one of the most widespread problems in cosmetic disorders which can cause psychological dysfunctions because of affecting from self-perception and judgment by others [1]. Although there is no morbidity associated with infra orbital dark circles, they are gaining more attention in society as a well-being concern. IOD can be observed based on person's appearance and some important parameters such as age and degree of fatigue [2]. It can occur in both sexes and all races, but more frequently in females and generally affect color patients more than Caucasians. This complex multifactorial entity needs to expanding knowledge base to understand etiology, management, and treatment [3]. As an important cosmetic concern, IOD can even influence self-confidence, well-being, and quality of life, particularly in female patients. Despite global prevalence of IOD, there is few studies and investigations on the dark circle, including its pathogenesis, treatment, and burden [4].

Although IOD is mostly known as a non-pathological situation, it can be related to serious pathogenesis as well. The main etiologies, which explain the occurrence of this complicated entity, are hereditary or environmental melanocytosis; for example, sunlight exposure, side effect of medications allergic or atopic dermatitis as post-inflammatory hyperpigmentation. Besides, IOD is increasingly prevalent in the elderly. In general, there is no clear etiology which is related to cause of IOD [5].

There are various cosmetic, radiofrequency, surgical, and laser treatments to ameliorate this condition [5][6]. None of the invasive and non-invasive therapies can provide effective treatment yet, consequently, the satisfaction and cure of patients is not satisfied. Therefore, new approaches and finding new- medications for IOD are welcome [7][8].

Traditional medicines and natural items have been suggested as possible sources of novel pharmaceuticals [9][10]. Persian medicine (PM), which dates back about 7,000 years [11][12][13], is one of the most well-known and oldest procedures among traditional and supplementary medical systems. IOD is described in PM sources. It was named Kamnat al dam in this traditional medical system [14].

Razes (854-925), one of the most prominent Persian scientists recommends topical use of zamad of Artemisia absinthium L. (Afsantin in Persian language) for IOD in Liber continents (Al-Havi), his comprehensive medical book. Despite no direct investigations on the effect of A.absinthium on IOD, current literature supports its probable effects. For example, current investigations approve the antioxidant effect of A.absinthium extract [15][16][17] that can play a key role to effect on IOD. Zamad (salve] is an ancient dosage form, which was made of a decoction mixture of grinded medicinal herbs. It currently has insufficient patient compliance. As a result, it is required to reformulate it into a popular formulation in order to improve product quality and patient compliance. Because of Non-popular form of this traditional formulation, an eye cream including an aqueous extract of A.absinthium was prepared. Various concentrations of extract were previously tested. Ultimately, 10% A.absinthium extract eye cream, as the best choice, was selected to include in this clinical trial. We aimed to evaluate the efficacy of this product as a clinical trial.

## Materials and Methods

Study Design

This double-blind, controlled clinical trial investigated the effect of Artemisia absinthium L. Eye-Cream on patients with Infra-Orbital Dark Circle who referred to Dermatology and Leprosy Research Center of Tehran University of Medical Sciences clinic from 1st October 2019 to 1st December 2019. Based on Inclusion criteria enrolled in this study.

Inclusion and Exclusion Criteria

All volunteer patients aged 18 to 65 years who had dark circles under their eyes at least for six months were included in the study, according to the inclusion criteria. The patients during breastfeeding or pregnancy, the patients using any chemical peeling procedures, performing microdermabrasion in the last 3 months, doing lasers in the last three months, doing a solarium or intense sun exposure and sunburn during the last three months, taking medicine due to other diseases, intolerance to the drug, reluctance to continue treatment and taking oral contraceptive pills while studying were excluded from this study.

Sample Size Calculation, Randomization, Allocation Process, Blinding

All participants were allocated into two groups of drug and placebo randomly via computer generated block-randomization list (non-stratified with equal-length blocks). The sample size was calculated by the below formula. Also, there were 5 more patients for each group (a total of 30 patients in each placebo and drug groups) for probable missing or excluded enrolled patients in both groups.

**Figure:**
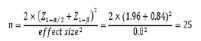


In this double-blind study, neither the patients and medical team nor the statisticians knew which group was receiving the drug or placebo.

Intervention

The study is a double-blind, controlled, randomized clinical trial. The participants (60 patients, 18-65 years old) were entered into two placebo and drug groups. Physicians, statisticians, and patients were unaware of the cream type. For this study, 60 healthy female and male volunteers with IOD were divided into two groups of 30. The cream was applied twice a day to the area around the eyes in each group for 60 days. By ending the period, the improvement in the intensity and appearance of dark circles of the participants were analyzed by a spectrophotometric colorimeter adapted for reflectance (Color Guide Sphere; Byk Gardner, Geretsried, Germany) and a Mexameter® (Courage and Khazaka, Koln, Germany) instruments. In this method, Mexameter® measured skin pigmentation (Melanin) and Erythema for investigation of drug effect. The infra-orbital and adjacent (cheeks) areas were selected for measurements by the probes, before and after the 60-day treatment (D1⁄T0 and D60). So, to evaluate the efficacy we used the color difference (ΔE) between the dark circles and the normal skin tone, the difference of lightness between dark circles and native skin tone (ΔL).

Ethical Issues

This study was approved by the Ethics Committee of Tehran University of Medical Sciences (no. IR.TUMS.VCR.REC.1398.017). Moreover, the trial registration number was obtained from the Iranian Clinical trial registration center, no. IRCT20190413043259N1. All patients were included in the study after being completely informed about the study and signing an informed consent form.

Chemical Substances

The chemical substances were provided from Sigma-Aldrich® (St. Louis, Missouri, United States). These includes Methanol High-Performance Liquid Chromatography (HPLC) grade, Acetonitrile HPLC grade, paraffin oil, cetyl alcohol, Tween 80, aspan 20, carbomer, sepigel, methylparaben, propylparaben, propylene glycol, NaOH, Na2HPO4, NaH2PO4, pbs.

Preparing Cream and Placebo

The aerial part of the plant Artemisia absinthium L. was collected from Gaduk passes of Mazandaran province in the north of Iran. The plant was approved by the Herbarium center in the School of Pharmacy, Tehran University of Medical Sciences (voucher number: 6604). Then, the freshly collected plant was washed and dried. The next step was the extraction process. In brief, 100 g of the powdered plant was placed in an extraction bottle and then was soaked with 1L of distilled water for 30 min at 100 °C and 180 rpm rotation. The extract was filtered and centrifuged at 4000 rpm (for 10 min) and stored at 4 °C. The cream was made based on an aqueous extract. Components and their weight percentages are shown in Table-1. The placebo cream was made based on Osirian without extract and the brown color (Khat e Zard brand, made in Iran, No. 110) was used to make a similar color to the drug. Moreover, a small amount of A.absinthium essential oil was rubbed on the placebo cream cap to make a similar smell to the drug.

**Table:** Table1. Components and Their Weight Percentage of Formulated Cream

**No.**	**Component**	**Weight percentage ** **(** **w/w%** **)**
**1**	Paraffin oil	5
2	Cetyl alcohol	5
3	Tween 80	0.5
4	Span 20	0.5
5	Carbomer	0.8
6	Sepigel	10
**Liquid phase **
1	Methylparaben	0.18
2	Propylparaben	0.02
3	propylene glycol	5
4	Aqueous extract	20
5	Water	Up to 100

Standardization of the A.absinthium Cream via the HPLC Method

An Agilent Technologies 1260 Infinity Π apparatus was used for HPLC analysis. for the quantification of Artemisinin, it was attached to an HPLC column Eclipse Plus-C18 (Agilent), 3.5 μm column (4.6×100 mm). The isocratic mobile phase of water, methanol, and acetonitrile (40:30:30% v/v) was used at a flow rate of 1.0 ml/min. UV monitoring was carried out at 210 nm. All solutions were filtered through a 0.45 mm filter before injection. Data analysis was performed using Agilent ChemStation software (Agilent, USA). The six concentrations of Artemisinin (5, 10, 30, 50, 70, and 100 μg ml-1 for Artemisinin) were used to generate a calibration curve of standard Artemisinin.

Dried extract or prepared cream was sonicated (15 min) in 2.5 mL of methanol. Centrifugation at 3300rpm was then used (5 min). A 10 mL volumetric flask was used to collect the supernatant. The aqueous extraction and formulated cream were injected into an HPLC apparatus to determine the quantified amount of Artemisinin in the preparation.

Investigation of Toxic Material via GC-MS Method

Thujone is a neurotoxic terpene substance found in an infamous plant such as A.absinthium [18]. Therefore, gas chromatography connected to mass detector (GC/MS) device was used to check for the presence of Thujone in the extract. The analysis of the aqueous extracted essence was performed using an Agilent 7890B GC, equipped with an HP-5MS capillary column (30 m, 0.25 mm i.d., 0.25 μm film thickness) and connected to a mass spectrometer 5977A as a detector. Helium was used as the gas carrier, with a flow rate of 1.5 mL/min. The column temperature was initially 30 °C (5 min), then gradually (5 °C/min) increased to 200; finally increased (10 °C/min) to 300 °C. GC-MS detections was carried out via an electron ionization system with an ionization energy of 70 eV. Then, diethyl ether was used to dilute the extract (sample) 1:100 (v/v), and 1.0 μL of it was injected into the apparatus automatically in splitless mode; with an injector temperature of 300 °C.

Preparation and Standardization of Drug and Placebo

**Figure-1:**
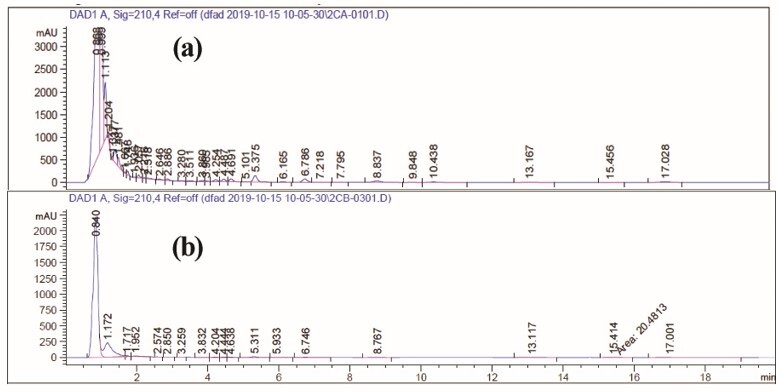
Figure1. High performance liquid chromatography (HPLC) chromatograms of a) aqueous extract of Artemisia absinthium L. and b) formulated cream.

Both drug and placebo creams were quite similar in appearance, color, and smell. Figure-1 shows HPLC chromatograms of the a) aqueous extract of A.absinthium and b) formulated cream of the standardization. The amount of Artemisinin in the plant extract and formulated cream were quantified at 4.33±0.02 μg/mg and 1.29±0.02 μg/mg, respectively.

As shown, the resulting extract is composed of 20 chemicals and the extract does not contain thujone which causes toxicity in the compounds. Therefore, the extract is safe for further research and preparation of the cream.

Statistics

In this section, the data were arranged and compiled based on the research objectives. The Shapiro-Wilk normality test was used for analyzing the normality of data. An independent t-test was used to evaluate the similarity of the two groups based on quantitative variables. The Chi-square test or Fisher’s exact test was used for qualitative variables.

Parametric analytical tests (paired t-test and independent t-test) were used for normally distributed data according to these tests, and non-parametric tests were used for non-normally distributed data according to these tests (Using IBM® SPSS® Statistics for Windows, version 22 (IBM Corp., Armonk, N.Y., USA)).

## Results

**Figure-2:**
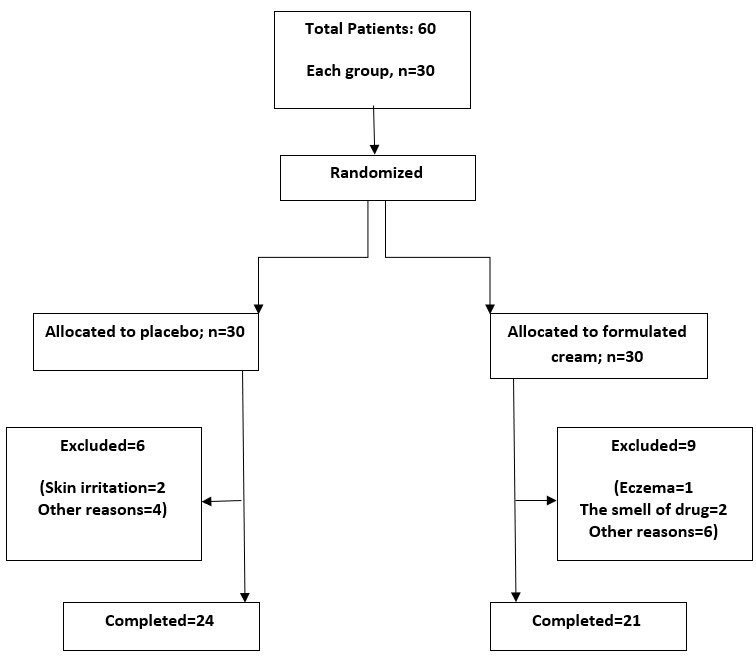
Figure2. CONSORT flowchart of participants in the study in two groups.

**Figure-3:**
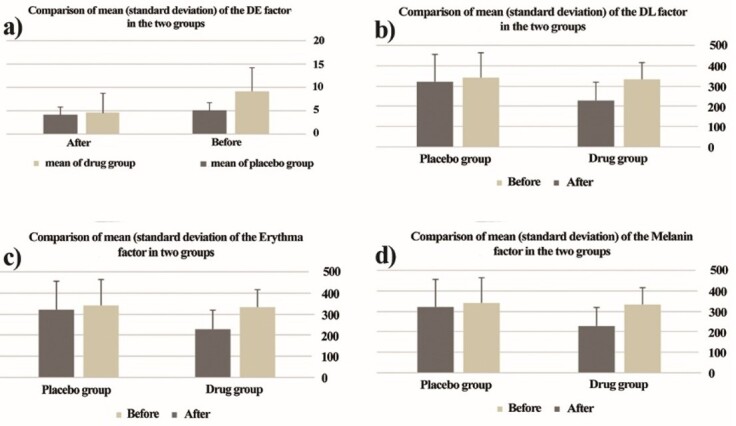
Figure3. Comparison chart of mean (SD) of **a):**delta erythema (DE); **b):** delta luminance (DL); **c):** erythema and **d):** melanin factors before and after research in two groups.

According to the demographic data in the baseline, there was no significant difference between the two groups (P=0.75) and in the percentage of female participants in the both groups (P=0.19, Table-2) as shown in the CONSORT flowchart (Figure2), among a total number of 60 patients, 45 (placebo group: 24 patients and drug group: 21 patients) completed the therapeutic protocol. The results of paired t-test in Placebo and Drug Groups are shown in Table-3 and -4. Table-5 shows the enrollment of patients in the study. The Melanin, Erythema, ΔE and ΔL of 21 volunteers in the drug group and 24 volunteers in the placebo group were measured. After that, they were treated with a designed cream and a placebo, and the desired parameters in both groups were tested again. The data was then evaluated using statistical techniques in the next stage. Since the assumption of normality of quantitative characteristics is required in samples less than 30, this default is evaluated in Table-5 by the Shapiro-Wilk test. Considering the significance level of the Shapiro-Wilk test shown in Table-2 for all factors in both groups before and after the research is greater than 0.05 (except Melanin is less than 0.05 and greater than 0.01 before the research in the placebo group), we concluded that the distribution of the above factors is not significantly different from the normal distribution. Therefore, the normal default is considered for performing statistical tests. As shown in Table-6 in the drug group, the difference of the mean (SD) ΔE , ΔL, Erythema and Melanin factors before and after the research were significant using paired t-test (P<0.001, P<0.001, P<0.002 and P<0.001, respectively) also in the placebo group were significant with paired t-test (P=0.001). As shown in Figure-3a-d, the decreased rate of ΔE, Erythema, and Melanin and the increase of ΔL in the drug group were more than in the placebo group, respectively. The results of the independent t-test showed that the mean value of ΔE and ΔL factors in both groups before the research were significantly different (P<0.001), but after the research between the two groups did not show a significant difference (P=0.46 and 0.49, respectively). The results of the independent t-test show that the mean value of the Erythema factor in the two groups both before (P=0.25) and after (P=0.5) between the two groups did not show a significant difference. The results of the independent t-test showed that the mean value of the Melanin factor in the two groups before

**Table:** Table2. Demographic Character of Participants in Treatment and Placebo Groups

**Variable(quantitative)**	**Treatment**	**Placebo**	***p*** **-Value**
**Age (year) Mean±SD**	41.43 ± 10.08	40.57±1.72	0.75
**Gender (F/M)**	29/1	25/5	0.19

**SD:**Standard deviation

**Table:** Table3. Paired t-test in Placebo Group

Parameters	Before	After	paired t-test
	mean±SD	mean±SD	
**ΔE**	5.07±1.63	4.14±1.58	0.001>
**ΔL**	-4.57±2.35	-3.44±2.26	0.001>
**Erythma**	414.64±70.31	369.93±61.90	0.001
**Melanin**	342.36±124.24	321.92±132.97	0.001>

**ΔE:**the color difference between dark area and normal skin tone;**ΔL:**the light difference between dark area and normal skin tone.

**Table:** Table4 Paired t-test in Drug Group

Parameters	Before	After	paired t-test
	mean±SD	mean±SD	
**ΔE**	9.23±3.31	4.57±2.23	0.001
**ΔL**	-7.89±3.39	-2.91±2.91	0.001>
**Erythma**	446.11±94.17	355.83±74.63	0.002
**Melanin**	334.56±82.19	229.86±87.09	0.001>

**ΔE:**the color difference between dark area and normal skin tone;**ΔL:**the light difference between dark area and normal skin tone.

the research was not significantly different (P=0.80), but after the research between the two groups showed a significant difference (P=0.01). Based on the results of paired t-tests, it can be determined that all variables have changed substantially in both groups. The medication group, on the other hand, faced a faster rate of change. But, the results obtained from the independent t-test in comparing the two groups before the test showed that both groups are comparable just in two factors, Erythema and Melanin (P>0.05) and in the two factors ΔE and ΔL before the experiment, the two groups have significant differences (P>0.05). As a result, comparing two factors ΔE and ΔL using the independent t-test, regardless of the difference in their initial values, is not without drawbacks. According to the results, it can be concluded that except for the Erythema factor, all other factors after the research was significantly different from the placebo group. This means that ΔE decreased, ΔL increased, and Melanin showed a significant increase in the drug group.

**Table:** Table5. Shapiro-Wilk Test for Normality of Research Factors before and after the Research in the Drug and Placebo Groups

		Drug	(n=21)	placebo	(n=24)
		t	Sig. (t-tailed)	t	Sig. (t-tailed)
Before	ΔE	0.95	0.39	0.94	0.20
Intervention	ΔL	0.95	0.37	0.92	0.055
	Erythem	0.94	0.26	0.95	0.28
	Melanin	0.92	0.10	0.91	0.036
After	ΔE	0.93	0.16	0.96	0.34
Intervention	ΔL	0.96	0.53	0.94	0.17
	Erythem	0.97	0.64	0.97	0.58
	Melanin	0.96	0.55	0.92	0.054

**ΔE:**the color difference between dark area and normal skin tone;**ΔL:**the light difference between dark area and normal skin tone.

**Table:** Table6. Comparison of Mean (SD) of Melanin, Erythema, DL and DE Factors before and after the Research in Two Groups

Parameters	Before		Independent t-test		After	Independent t-test
	Drug	Placebo	Sig	Drug	Placebo	
	mean±SD	mean±SD		mean±SD	mean±SD	
**ΔE**	9.23± 3.31	5.07±1.63	0.001>	4.57±2.23	4.14±1.58	0.46
**ΔL**	-7.89± 3.39	-4.57±2.35	0.001>	-2.91±2.91	-3.44±2.26	0.5
**Erythma**	446.11±94.17	414.64±70.31	0.21	355.83±74.63	369.93±61.90	0.49
**Melanin**	334.56±82.19	342.36±124.24	0.81	229.86±87.09	321.92±132.97	0.002

**ΔE:**the color difference between dark area and normal skin tone;**ΔL:**the light difference between dark area and normal skin tone.

## Discussion

Although IOD, as the darkness of the infra-orbital eyelids is not identified as a medical concern, it is highly prevalent and can be a cosmetic concern. On the other hand, unfortunately, few published articles are about the dark circles’ issue [1][19]. This problem occurs by various factors, such as the presence of excessive pigment, thinness, and clarity of the skin below the eyelid and shading due to looseness and tearing of the skin [20]. Unfortunately, existing treatments for dark circles, such as lasers, injections, fillers, and chemicals, have several issues and adverse effects [21][22][23][24].

Herbal medications are the most promising complementary and alternative therapy being among patients, according to studies [25], although these studies were restricted. Therefore, we investigated the effect of a cream prepared from the aqueous extract of Artemisia absinthium L., which was used in Persian medicine for the treatment of dark circles under the eyes.

The main purpose of this investigation was to analyze the effectiveness of eye cream prepared from an aqueous extract of A.absinthium on dark circles around the eyes.

The results of this study showed that treatment of dark circles with herbal cream for 8 weeks (2 months) can be effective except for the Erythema factor. In this study, the ΔE decreased, ΔL increased, and Melanin showed a significant decrease in the drug group.

Generally, the rate of improvement in patients with dark circles in the group receiving herbal cream based on the indicators has significantly increased without any side effects. The antioxidant effect is a key point for managing IOD.

Numerous studies have confirmed the existence of free radical scavenging properties in the total extract of Artemisia absinthium L. [26][27]. Rashidi et al. showed that the extracts of Artemisia absinthium L. can prevent the damaging effects of oxidative agents on cells [28]. Furthermore, Kharoubia et al. suggested that wormwood extract restored enzyme activities perturbed by exposure to lead; therefore, wormwood extract had a protective role against lipid peroxidation [29]. According to studies, oxidative damage could be occurred due to the formation of reactive oxygen species (ROS). It leads to the deterioration of cellular macromolecules like lipids, deoxyribose nucleic acids (DNA), proteins, and enzymes [30][31].

On the other hand, a study showed that extracts with free radical scavenging properties can reduce Melanin and skin pigmentation [32]. As a result, the characteristic of free radical scavenging of the Artemisia absinthium L. extract may be linked to the reduction in Melanin seen in those who have used the medicinal cream.

Moreover, another study showed that the extract of this plant increased blood circulation, which can be attributed to the increased Erythema [33].

## Conclusion

Despite the widespread use of various methods of treating dark circles under the eyes, many people today suffer from this problem. On the other hand, these treatments, such as exfoliators, chemicals, lasers, etc., have many side effects for patients.

As a result, using traditional Persian medical remedies might be one of the most promising solutions to this condition. Unfortunately, there is very little study on the use of herbal medicines to address this condition. The present study is the first study which investigated the effect of A.absinthium in the treatment of this problem.

In general, based on the results of this research, the cream prepared from this extract of the plant can be suggested as an effective and safe treatment for removing dark circles.

## Acknowledgments

This paper is a result of PhD thesis of Hanan Hamdi, supported by the vice chancellor for research and technology at Tehran University of Medical Sciences (Code: 9542711002) and presented in the School of Persian Medicine at Tehran University of Medical Sciences.

## Conflict of Interest

There is no conflict of interest.
